# Implementing the Brazilian Database on Orofacial Clefts

**DOI:** 10.1155/2013/641570

**Published:** 2013-03-12

**Authors:** Isabella Lopes Monlleó, Marshall Ítalo Barros Fontes, Erlane Marques Ribeiro, Josiane de Souza, Gabriela Ferraz Leal, Têmis Maria Félix, Agnes Cristina Fett-Conte, Bruna Henrique Bueno, Luis Alberto Magna, Peter Anthony Mossey, Vera Gil-da Silva-Lopes

**Affiliations:** ^1^Medical Genetics Sector, State University of Alagoas (UNCISAL), Brazil; ^2^Clinical Genetics Service, Federal University of Alagoas (UFAL), Brazil; ^3^Medical Genetics Sector, Hospital Infantil Albert Sabin (HIAS), Brazil; ^4^Medical Genetics Sector, Assistance Center for Cleft Lip and Palate (CAIF), Brazil; ^5^Medical Genetics Sector, Facial Deformity Care Center (CADEFI), Brazil; ^6^Medical Genetics Service, Hospital de Clínicas de Porto Alegre (HCPA), Brazil; ^7^Molecular Biology Department, Medicine School of São José do Rio Preto (FAMERP/FUNFARME), Brazil; ^8^Department of Medical Genetics, Faculty of Medical Sciences, University of Campinas, 13083-887, Brazil; ^9^Dundee University Dental School, UK

## Abstract

*Background*. High-quality clinical and genetic descriptions are crucial to improve knowledge of orofacial clefts and support specific healthcare polices. The objective of this study is to discuss the potential and perspectives of the Brazilian Database on Orofacial Clefts. *Methods*. From 2008 to 2010, clinical and familial information on 370 subjects was collected by geneticists in eight different services. Data was centrally processed using an international system for case classification and coding. *Results*. Cleft lip with cleft palate amounted to 198 (53.5%), cleft palate to 99 (26.8%), and cleft lip to 73 (19.7%) cases. Parental consanguinity was present in 5.7% and familial history of cleft was present in 26.3% subjects. Rate of associated major plus minor defects was 48% and syndromic cases amounted to 25% of the samples. *Conclusions*. Overall results corroborate the literature. Adopted tools are user friendly and could be incorporated into routine patient care. The BDOC exemplifies a network for clinical and genetic research. The data may be useful to develop and improve personalized treatment, family planning, and healthcare policies. This experience should be of interest for geneticists, laboratory-based researchers, and clinicians entrusted with OC worldwide.

## 1. Introduction

Accurate and detailed phenotype description of orofacial clefts (OC) is crucial to produce good etiological and epidemiological studies. In this regard, attention should be given to subphenotypic features of the lip (completeness of the cleft, presence of pits/prints, dental and orbicularis oris muscle anomalies), and palate (completeness of the cleft, submucous defects, bifid uvula, and ankyloglossia). Similarly important is the screening of minor and major associated defects which has prevalence rate that ranges from 8% to 75%. Although there are true population differences, methodological factors such as sample source and size, method of ascertainment, case definitions, inclusion criteria, coding system, and case classification account for much of this wide variation [1–13].

In the postgenomic era capturing and processing information on human genetic variation, gene-environment interactions, and genotype-phenotype correlations are essential to develop personalized interventions. This has been reinforced by the Human Variome Project (HVP), an international effort launched in 2006. The HVP aim is to develop and make knowledge housed within linked databases on genes, mutations, and variants accessible to the research and medical communities [14, 15].

Databases may also serve as tools in educating health professionals, policymakers, and the general public towards prevention of unnecessary suffering, improvement of healthcare, and elimination of erroneous beliefs that still remain in some cultures. On humanitarian and ethical grounds, these should be ultimate reasons for birth defects research [16–19].

Recognizing the impact of OC, the World Health Organization assigned the coordination of the International Perinatal Database of Typical Orofacial Clefts (IPDTOC) to the International Clearinghouse for Birth Defects Surveillance and Research (ICBDSR), in 2002 [18–20].

As stated in the report *Global Registry and Database on Craniofacial Anomalies* [18], the quality of recorded data should be of more concern than completeness of ascertainment in this kind of system. The IPDTOC was launched in 2003 and has collected and analysed case-by-case clinical and epidemiological information OC from birth defects registries worldwide using a standard definition and system for case classification [12].

Care for people with OC in Brazil has been funded by the government through the Unified Health System (Sistema Único de Saúde, SUS) since 1993. In 1998, the National Health Ministry (NHM) created the Brazilian Reference Network for Craniofacial Treatment (RRTDCF). These measures, however, were not preceded or followed by assessment of specific characteristics and impact of OC on the Brazilian population. Currently the RRTDCF numbers 21 units, but just five of them count with geneticist/dysmorphologist in the team [17, 21, 22].

Brazil is a continental country of more than 180 million inhabitants with diverse genetic background and multicultural profile. There is still a shortfall in epidemiological data on overall birth defects in the country. Similar to other parts of the world, data recorded through birth certificates has been criticized on the grounds of ascertainment, sensitivity, reliability, completeness, and consistence of the reports [23–25].

According to the Latin-American Collaborative Study of Congenital Malformations (ECLAMC), the Brazil's birth prevalence of cleft lip (CL) is 49/10,000, cleft lip with cleft palate (CLP) is 116/10,000, and cleft palate (CP) is 58/10,000 [26]. ECLAMC is a hospital-based register which covers only 2% of all Brazilian births [25]. Despite this limitation, the high quality of ECLAMC's data and the fact that OC are among the best ascertained birth defects probably make these figures representative of Brazil's prevalence.

Besides ECLAMC, some cleft services and hospitals linked or not to the RRTDCF record data according to their research field of interest. Therefore, they may include epidemiological, morbidity, mortality, clinical, genetic, and outcome issues. Information gathered, however, is not standardized [17].

High quality of clinical and genetic descriptions is crucial to improve knowledge on OC and support specific healthcare polices. The development and implementation of the Brazilian Database on Orofacial Clefts (BDOC) reported here is a pioneer nationwide initiative to fill in the gap on clinical and genetic information on OC in the Brazilian population.

## 2. Aim

The aims of this study are to report the implementation, to describe preliminary clinical and familial characteristics and to discuss the potential, and perspectives of the BDOC. 

## 3. Methods

### 3.1. Database Design

BDOC is a nationwide, hospital-based, voluntary and primary database. Initially, a 10-year schedule was planned to run as a continuous and flexible system in which new aims and tools can be aggregated according to the experience gained. General planning of activities started in 2006 and the validation of the tools started in 2008 in voluntary hospitals with clinical geneticists. According to the strategy originally proposed, after this phase, other hospitals without geneticists could be invited to participate. Clinical and laboratory data are updated during the followup of each subject. The database was approved by the local Institutional Review Boards and the National Research Ethics Committee (CONEP # 14733). All subjects provided informed consent.

Standardized individual and familial information forms the core database. It is complemented by other five satellite protocols designed to cover the following issues: (1) genetic (Biobank of DNA); (2) morbidity and mortality; (3) services' structure and dynamics; (4) professionals' educating characteristics and protocols; and (5) subject/parent satisfaction. 

Core database was initiated in eight sites comprising three units of the RRTDCF, two multiprofessional non-RRTDCF centres, and three genetic services. These sites were invited because they all have geneticists with clinical experience in dysmorphology. All of them were personally visited by the coordinators (ILM and VLGSL) before starting the collection of data. 

### 3.2. Target Population, Inclusion Criteria, and Work Definitions

Individuals with typical OC and Pierre-Robin sequence in isolated and nonisolated presentation were included. Data on abortuses, stillbirths, cleft uvula, median, oblique, and submucous clefts were not included.

Typical OC (CL, CLP, and CP) and Pierre-Robin sequence were defined according to the International Classification of Diseases 10th Edition. Terms *isolated* and *associated* were used to refer to additional minor or major defects regardless of the cause or mechanism involved while *syndromic* and *nonsyndromic*, to refer to the underlying aetiology [13].

Case classification was based on the definitions of the IPDTOC Working Group (2011) which defines three phenotype categories: isolated clefts, recognized syndrome, and multimalformed cases (MMC). Accordingly, cases of known nonrandom association (e.g., VACTERL) are included into the category of recognized syndromes. Cases with random combination of major unrelated defects with evidence of distinct aetiological factors are included in the group of MMC. Deformities were considered minor defects [12]. A list of minor defects was reviewed along with ICBDSR in May 2007 and is available at http://www.icbdsr.org/. 

### 3.3. Collection, Storage, and Processing of Data

Data were collected using a paper-based record form specifically designed for this database according to the operating manual. These tools were based on the “US National Birth Defects Prevention Study” and ESF “common core protocols” for cleft research and developed as part of a previous study [27].

The record form comprises 80 questions which cover the following information: (1) obstetric, birth, neonatal, and medical history; (2) socioeconomic status; (3) family history where possible (1st-, 2nd-, and 3rd-degree relatives); (3) type of cleft according to topography (lip, alveolus, hard, and soft palate), severity (unilateral or bilateral) and laterality (left and right sided), (4) morphological assessment (including verbatim description of dysmorphic features); (5) laboratory tests (standard cytogenetics, fluorescence in situ hybridization—FISH) and/or molecular analysis, biochemical tests, ultrasound, X-ray images, and so forth; and (5) diagnosis and its evidences. 

Operating manual includes the following content: (1) diagram for phenotype categorization, (2) operating definitions, (3) clinical descriptors, (4) list of related defects, (5) list of minor defects, (6) examples of twinning, (7) examples of toxic and occupation-related substances, (8) examples of consanguineous relationships, (9) instructions for taking standard photographs, and (10) examples on how to fill the form.

All subjects were personally interviewed and examined by the participant geneticist during routine genetic evaluation from November 2008 to December 2010. As the major proposal of this database is to collect clinical and familial information, there were no restrictions regarding age and existence of previous cleft surgery at the time of enrolment.

Forms were sent by post or delivered in person for data reviewing and coding at Unicamp where the BDOC is seeded. The data manager checked all the information received and sought for clarifications when needed. Before entering data into the electronic database, all record forms were manually reviewed and coded by the coordinators. 

### 3.4. Pretest of the BDOC Tools and Strategy for Group Management

Record form and operating manual were pretested by seven geneticists throughout a six-month period. A total of 143 record forms and 10 assessment questionnaires were included. Mean time spent to complete the record form at the end of this phase was 20 minutes (SD = 4.57) [28].

All geneticists asked for revision of wording, spacing, and ordering of some questions. Coordinator centre asked for further information and clarification of some responses in 28 record forms. Despite this, record form and operating manual were assessed as useful and reliable tools [28].

At the end of this phase a unit of the RRTDCF ceased its participation and, a new site, a genetic service, was included. All participants attended biannual meetings to exchange experience, discuss data, and plan the next stages. 

### 3.5. Statistical Methods

The electronic database was built using Microsoft Access version 2007. Data processing and analysis were performed using two statistical packages (SPSS for Windows version 15.0 and EpiInfo versions 3.5.1 and 3.04d). Categorical variables were analysed using chi-square test. Numerical variables were tested for normality using one-sample Kolmogorov-Smirnov test. Variables with normal distribution were tested using ANOVA and Student's *t*-test, while variables with nonnormal distribution were tested using Kruskal-Wallis and Mann-Whitney tests. The significance level of 5% (*P* < 0.05) was adopted for all tests.

## 4. Results

**Figure 1:**
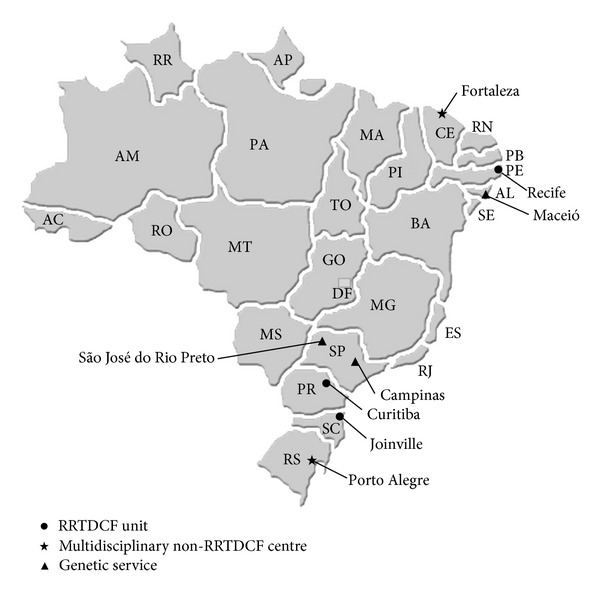
Map of Brazil showing localization of cities and sites participating in the Brazilian database on orofacial clefts (BDOC).

**Figure 2:**
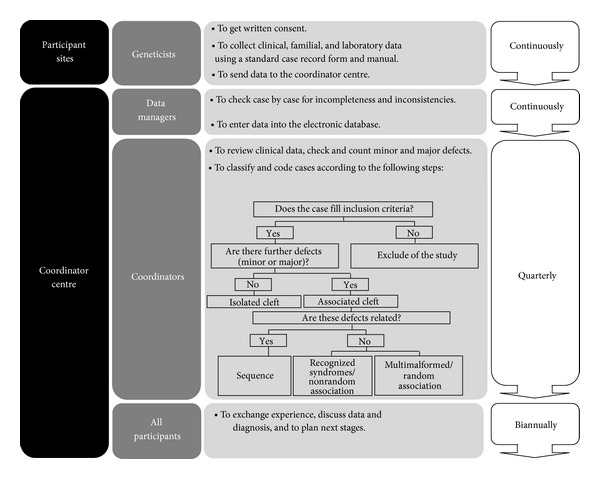
A summary of the process of recording cases through the Brazilian database on orofacial clefts (BDOC). Please refer to text for details.

Demographic, clinical, and familial information of 370 individuals with OC was prospective and systematically recorded in the sites identified in Figure 1. As only minor amendments were recommended after the testing phase, respective record forms were included into the sample.  Figure 2 summarizes how the BDOC worked throughout the studied period.

Majority of subjects (63.2%) were living in the northeast of Brazil. Males amounted to 219 (59.2%) while females to 151 (40.8%). Birth weight was available in 324 cases. It ranged from 1195 g to 4900 g (mean = 3100; mode = 2800; median = 3100; and SD = 606.6). Age ranged from 0 to 50 years (mean = 4.3; mode = 0; median = 0.5; and SD = 7.3).

Table 1 shows the distribution of patients according to participant site, geographic origin, age, and birth weight at the time of enrolment in the database. Ratio of patients seen at units of the RRTDCF, cleft multidisciplinary non-RRTDCF centres, and genetic services was 1.3 : 1.0 : 1.1. Patients seen at genetic services were significantly older (*P* < 0.001) and had lower birth weight (*P* = 0.047) than those seen elsewhere.

Distribution of subjects according to type of cleft (CL, CLP, and CP) with regard to gender, severity, laterality, and presence of additional defects is shown in Table 2. CLP prevailed over CP and CL, unilateral over bilateral, and left- over right-sided clefts.

Chi-squared contingency table revealed significant differences among all groups of clefts. There was an excess of males among individuals with CLP + CL (male : female ratio = 1.85, *P* = 0.0001) and of females among those with CP (female : male ratio = 1.30, *P* = 0.0001). Bilateral clefts were more frequent for subjects with CLP than for those with CL (*P* = 0.0002). 

Parental consanguinity was present in 21/368 (5.7%) cases, 10 of which were first cousins. Consanguineous marriages were not statistically associated with geographic origin (*P* = 0.425), type of cleft (*P* = 0.451), and phenotype category (i.e., if isolated versus syndromes and MMC) (*P* = 0.381). 

Excluding 12 individuals from whom familial data were not available, familial history of cleft was found in 94/358 (26.3%) cases. First-degree relatives were affected in 18 (19.4%), second degree in 12 (12.9%), and third degree and above in 63 (67.7%) families. There was an excess of affected relatives in the CLP subgroup in comparison with CP (*P* = 0.005) but not with CL (*P* = 0.183).

Global rate of associated defects (minor plus major) was 179 (48.4%). Fifty-nine (15.9%) subjects showed both minor and major, 103 (27.8%) had only minor and 17 (4.6%) had only major-associated defects. As shown in Table 2, there was no statistically significant difference among subgroups of cleft with regard to rate of minor defects (*P* = 0.193). On the other hand, major defects were more likely to be found in the groups of CLP and CP and unlikely in the group of CL (*P* = 0.003).

Ninety-three (25%) subjects were classified as having syndromic clefts. Comparisons between nonsyndromic and syndromic cases are presented in Table 3. There were no statistically significant differences between these groups with regard to gender and severity of the cleft. 

Syndromic cases were more likely to be found at genetic services (*P* = 0.006) and statistically more associated with CP (*P* = 0.000). These cases also showed lower birth weight in both categorical (*P* = 0.001) and quantitative analyses (*P* = 0.000) and higher mean of *minor* defects (*P* = 0.000). 

**Figure 3:**
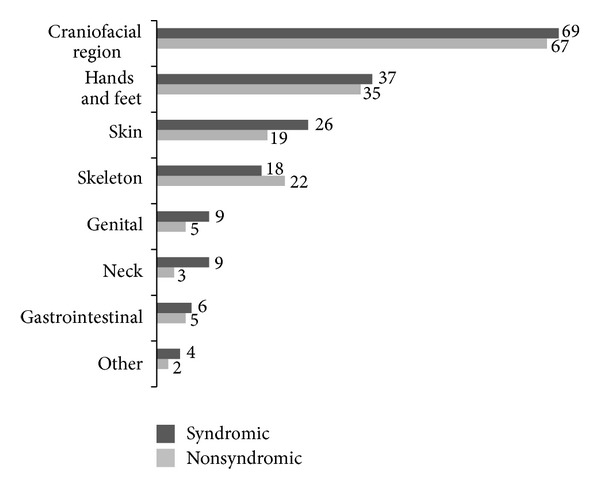
Distribution of minor defects according to anatomic region between nonsyndromic and syndromic cases.

**Figure 4:**
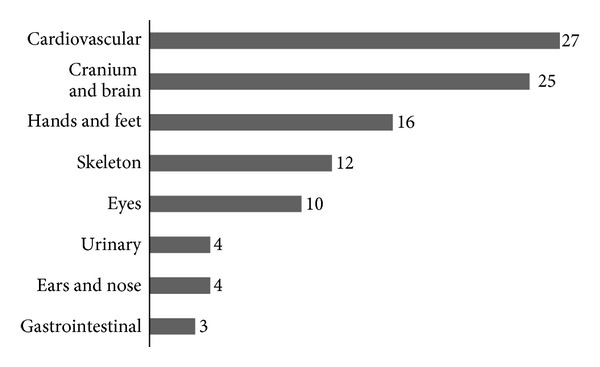
Distribution of major defects according to anatomic region among syndromic cases.

Subjects were regrouped according to the number of minor defects into two categories (1–3 and ≥4 defects). There was a predominance of syndromic cases in the subgroup with four and above *minor* defects (*P* = 0.000). Anatomic distribution of *minor* and *major* defects is shown in Figures 3 and 4, respectively. 

Based on verbatim description, the following phenotype categories were identified: 277 (75%) isolated clefts, 47 (12%) recognized syndromes or associations, and 46 (13%) multiple malformed cases (Table 3). Eighteen (40%) individuals with recognized syndromes had not additional *major* defects. 

The 47 clinical recognized syndromes and associations were categorized according to aetiology. Mendelian syndromes were in the lead (*n* = 21; 45%), followed by chromosomal (*n* = 18; 38%), heterogeneous (*n* = 6; 13%), and teratogenic categories (*n* = 2; 4%). 

## 5. Discussion

The BDOC was designed to gather detailed, high-quality, and continuing updated information on clinical and familial characteristics of OC in the Brazilian population. This is crucial to set a solid basis for future genotype-phenotype studies in which accuracy and consistency of the collected data are issues of much concern than level of ascertainment [18, 19].

In this study, information was prospectively collected by experienced geneticists during their ordinary activities. Data was recorded and processed following a standard method and a strictly defined protocol.

The database was regularly updated with new clinical or laboratory data of patients registered. If on one hand these are strengths of our database, on the other hand they make the process of record taking lengthy. Moreover, complementary investigation and genetic tests are not equally available in different regions of Brazil. These issues have direct implications on the number of cases we are able to record and follow per year and should be borne in mind when interpreting our results.

More than 84.3% of the Brazilian population lives in the three regions from which our data were collected. Among them, southeast is the most densely inhabited area followed by northeast and south [29]. These regions host 17 out of 21 units of the RRTDCF, 18 out of 22 multidisciplinary non-RRTDCF sites [17], and 47 out of 56 clinical genetic services [30] of the country. 

Subjects of this study were predominantly from northeast, followed by south and southeast. This result does not reflect differential prevalence of OC but specificities of the participant sites. Southeast region is represented by two genetic services in which individuals attend with various birth defects, OC included. South is represented by three sites specifically dedicated to cleft care (two RRTDCF units plus a multidisciplinary non-RRTDCF site). The northeast region counts with a site of each category of service (genetic, RRTDCF, and multidisciplinary non-RRTDCF). The genetic service from the last-mentioned region was conducting a parallel study on OC which justifies its high amount of subjects [31]. Taking these specificities into account, participant sites were proportionally represented in the study.

Study design allowed a wide range of subjects' age at enrolment. Younger individuals were preferentially seen in multidisciplinary non-RRTDCF and RRTDCF sites. Subjects enrolled at genetic units were among the oldest, suggesting that the subjects primarily refer to surgery treatment instead of genetic evaluation. In addition, previous studies [22, 31] have showed that there is high level of inequality to access genetic evaluation and counselling in Brazil.

The overall results on type of cleft, gender ratio differences, severity (unilateral and bilateral) and laterality (left and right sided) of the lip defects corroborate the literature [2, 11, 13, 32, 33].

Prevalence of consanguinity in this sample was higher than that reported for the Brazilian population [34–36] and did not show preferential geographic distribution. It has been suggested that there is a greater genetic component in the aetiology of CL based on the observation of an excess of individuals with CL over CLP in the offspring of consanguineous parents [37]. We did not find statistically significant association between type of cleft and parental consanguinity. This result, however, should be confirmed in the future using a larger sample.

In the present study, more than one in four subjects showed family history of OC and almost one in five had an affected individual among their first degree relatives. In this subgroup, CLP was the most prevalent followed by CL and CP. A population-based study conducted in Denmark showed that anatomical severity does have an effect on recurrence in first-degree relatives and the type of cleft is predictive of the recurrence type. Third-degree relatives also have an increased recurrence risk compared to the background population [38].

In our sample, global rate of associated defects was around 48% and predominated among subjects with CP. Minor defects were more prevalent in craniofacial region, while cardiovascular and central nervous systems were mainly and almost equally affected by major defects. One out of four individuals was assigned as a syndromic case. Proportion of syndromic cases was higher among individuals with CP and lower among those with CL. 

Methodological differences regarding case definition, inclusion or exclusion of minor defects, and anomalies/syndromes grouping-system hinder comparisons with many published data. Despite this, our results on global rate of associated defects and anatomic regions involved are similar to those reported by previous studies [9, 13, 32].

Among more than 20 investigated genes, *IRF6*, *VAX1,* and 8q24 *locus* have a confirmed role in nonsyndromic OC. Environmental factors, lifestyle, and the preventive role of vitamin supplements have been also investigated. Maternal smoking during pregnancy is consistently linked with increased risk of OC. Findings on the other risk factors and gene-environment interactions, including folic acid, have been inconclusive due to methodological issues [1–4, 39]. Besides these factors, a meta-analysis approach showed that parents of 40 years or older have higher probability of having a child with OC [40].

Despite important advances in the understanding of nonsyndromic OC, around 50% of patients with syndromic pictures remain as cases of multiple congenital anomalies without an identifiable aetiological factor. Laboratory facilities have improved the rate of specific diagnosis so that more than 600 syndromes involving OC have been already recognized. Chromosomal aberrations are the most frequent aetiological group, followed by Mendelian/heterogeneous abnormalities and teratogenic factors [8, 13, 32, 41, 42].

Numeric and structural chromosomal abnormalities, including 22q11 deletion, were detected in 13 cases. Mendelian, heterogeneous, and teratogenic conditions were diagnosed on the basis of clinical evidences. 

Limited laboratory facilities are challengeable and the Brazilian database may be helpful to define which tests are critical to our population. Collaboration to make these tests available into the network would be economically advantageous. This is an important strategy for healthcare planning [15, 43–45].

As knowledge of genetics and of gene-environment interaction in the aetiology of OC improves, clinical genetics is becoming increasingly important specialty to ensure accurate diagnosis and allow appropriate genetic counselling [3]. Therefore, clinical genetic approach improves accuracy, consistency, and reliability of clinical descriptions and aetiological assessment which are critical to genotype-phenotype correlations. Understanding these imbricate mechanisms using modern technologies is important to improve therapy and prevention. 

The process of interpreting clinical data to determine whether an individual has the defect of interest as syndromic or nonsyndromic defect is complex and involves some degree of subjectivity [4, 46]. Methods and terminology should be as well-defined as possible in order to make process uniform. In this regard, adoption of a stepwise approach is much advantageous [4].

The experience reported here shows how a group of geneticists has developed, implemented, and maintained a network suitable for clinical and genetic research on OC. Strengths of this study are that (1) information is prospectively collected by geneticists experienced in dysmorphology following a standard method and strictly defined protocol; (2) data is centrally storage and processed following a defined stepwise approach which uses IPDTOC/ICBDMS definitions, descriptors, and code system; (3) case record form and operating manual are user-friendly tools and may be incorporated in the routine in other cleft centres, these tools are available to interested researchers through contact with the Cranio-Face Brazil Project (cranface@fcm.unicamp.br); (4) experience gained throughout the process is shared among participants in face-to-face biannual meetings which improve enthusiasm and cohesiveness of the group.

Approaches with new technologies such as Genome Wide Association Studies (GWAS) and open array using accurate clinical data probably would bring interesting results to improve knowledge on the aetiology of OC. Besides research applications, information gathered in the BDOC may be useful to develop and improve personalized treatment, family planning, and public health policies on clinical and laboratory genetic investigation. This should be of interest not only for geneticists and laboratory-based scientists but also—and perhaps especially—for policymakers and clinicians entrusted with OC worldwide.

## Figures and Tables

**Table 1:** Distribution of subjects according to participant site, geographic origin, age, and birth weight.

	RRTDCF	Multidisciplinary non-RRTDCF	Genetic service	Total
Number of cases *n* (%)	141 (38)	107 (29)	122 (33)	370 (100)
Geographic origin				
Northeast *n* (%)	62 (44)	86 (80)	86 (70)	234 (63.2)
South *n* (%)	79 (56)	21 (20)	—	100 (27)
Southeast *n* (%)	—	—	36 (30)	36 (9.7)
Age (years, mean)^#^	2.7	1.5	8.5	4.3*
Birth weight (grams, mean)^†^	3,057	3,152	2,932	3,055**

^#^Mann-Whitney test: RRTDCF × non-RRTDCF, *P* = 0.049; RRTDCF × genetic services, *P* < 0.0001; non-RRTDCF × genetic services, *P* < 0.001; *Kruskal-Wallis test, *P* = .000; ^†^LSD test: RRTDCF × non-RRTDCF, *P* = 0.230; RRTDCF × genetic services, *P* = 0.136; non-RRTDCF × genetic services, *P* = 0.014; **ANOVA, *P* = 0.047.

**Table 2:** Distribution of subjects according to type of clefts with regard to gender, cleft's severity and laterality, and presence of additional defects.

Variable	CLP	CL	CP	Total	P^#^
Number of cases (%)	198 (53.5)	73 (19.7)	99 (26.8)	370 (100)	
Gender					0.0001
Male	137 (69)	39 (53)	43 (43)	219 (59)	
Female	61 (31)	34 (47)	56 (57)	151 (41)	
Severity					0.0002
Unilateral	120 (60)	62 (85)	—	182 (67)	
Bilateral	78 (40)	11 (15)	—	89 (33)	
Laterality					0.0001
Unilateral left	85 (70)	41 (66)	—	126 (69)	
Unilateral right	35 (30)	21 (34)	—	56 (31)	
Additional minor defects					0.193
Yes	87 (44)	26 (36)	49 (49)	162 (44)	
No	111 (56)	47 (64)	50 (51)	208 (56)	
Additional major defects					0.003
Yes	41 (21)	6 (8)	29 (29)	76 (21)	
No	157 (79)	67 (92)	70 (71)	294 (79)	

^#^Chi square.

**Table 3:** Distribution of syndromic and nonsyndromic cases according to several variables.

Variables	Nonsyndromic	Syndromic	Total	*P* ^#^
Number of cases (%)	277 (75)	93 (25)	370 (100)	
Category of site				0.006
RRTDCF unit	111 (40)	30 (33)	141 (38)	
Non-RRTDCF centre	87 (31)	20 (21)	107 (29)	
Genetic services	79 (29)	43 (46)	122 (33)	
Gender				0.324
Male	168 (61)	51 (55)	219 (59)	
Female	109 (39)	42 (45)	151(41)	
Birth weight (grams)				
≤2500	27 (11)	22 (26)	49 (21)	0.001
>2500	212 (89)	63 (74)	275 (79)	
*Mean *	3,145	2,800	3,055	0.000
Type of cleft				0.000
CLP	152 (55)	46 (49)	198 (53)	
CL	65 (23)	8 (9)	73 (20)	
CP	60 (22)	39 (42)	99 (27)	
Severity				0.167
Unilateral	150 (69)	32 (59)	182 (67)	
Bilateral	67 (31)	22 (41)	89 (33)	
Minor defects				0.000
0	192 (69)	16 (17)	208 (56)	
1–3	57 (21)	31 (34)	88 (24)	0.141
≥*4 *	28 (10)	46 (49)	74 (20)	0.000
Mean	2.97	5.09	3.98	.000
Major defects (OC excluded)				
0	277 (100)	19 (20)	296 (80)	—
1–3	—	69 (74)	69 (19)	—
≥4	—	5 (6)	5 (1)	—
Mean	—	1.59	—	—
Phenotype category				
Isolated cleft	277 (100)	—	277 (75)	—
Syndrome and association	—	47 (51)	47 (13)	—
Multimalformed case	—	46 (49)	46 (12)	—

^#^Chi square.

## References

[1] Dixon MJ, Marazita ML, Beaty TH, Murray JC (2011). Cleft lip and palate: understanding genetic and environmental influences. *Nature Reviews Genetics*.

[2] Mossey PA, Little J, Munger RG, Dixon MJ, Shaw WC (2009). Cleft lip and palate. *The Lancet*.

[3] Mossey PA, Shaw WC, Munger RG, Murray JC, Murthy J, Little J (2011). Global oral health inequalities: challenges in the prevention and management of orofacial clefts and potential solutions. *Advances in Dental Research*.

[4] Luijsterburg AJM, Vermeij-Keers C (2011). Ten years recording common oral clefts with a new descriptive system. *The Cleft Palate-Craniofacial Journal*.

[5] Shprintzen RJ, Siegel-Sadewitz VL, Amato J, Goldberg RB (1985). Anomalies associated with cleft lip, cleft palate, or both. *American Journal of Medical Genetics*.

[6] Milerad J, Larson O, Hagberg C, Ideberg M (1997). Associated malformations in infants with cleft lip and palate: a prospective, population-based study. *Pediatrics*.

[7] Croen LA, Shaw GM, Wasserman CR, Tolarova MM (1998). Racial and ethnic variations in the prevalence of orofacial clefts in California, 1983–1992. *American Journal of Medical Genetics*.

[8] Tolarova MM, Cervenca J (1998). Classification and Birth prevalence of orofacial clefts. *American Journal of Medical Genetics*.

[9] Beriaghi S, Myers S, Jensen S, Kaimal S, Chan C, Schaefer GB (2009). Cleft lip and palate: association with other congenital malformations. *Journal of Clinical Pediatric Dentistry*.

[10] Wyszynski DF, Sárközi A, Czeizel AE (2006). Oral clefts with associated anomalies: methodological issues. *The Cleft Palate-Craniofacial Journal*.

[11] Genisca AE, Frías JL, Broussard CS (2009). Orofacial clefts in the national birth defects prevention study, 1997–2004. *American Journal of Medical Genetics A*.

[12] Mastroiacovo P, Maraschini A, Leoncini E (2011). Prevalence at birth of cleft lip with or without cleft palate: data from the International Perinatal Database of Typical Oral Clefts (IPDTOC). *The Cleft Palate-Craniofacial Journal*.

[13] Rittler M, Cosentino V, López-Camelo JS, Murray JC, Wehby G, Castilla EE (2011). Associated anomalies among infants with oral clefts at birth and during a 1-year follow-up. *American Journal of Medical Genetics A*.

[14] Kaput J, Cotton RG, Hardman L (2009). Planning the human variome project. The spain report. *Human Mutation*.

[15] Patrinos GP, Aama JA, Aqeel AA (2010). Recommendations for genetic variation data capture in developing countries to ensure a comprehensive worldwide data collection. *Human Mutation*.

[16] Botto LD, Robert-Gnansia E, Siffel C, Harris J, Borman B, Mastroiacovo P (2006). Fostering international collaboration in birth defects research and prevention: a perspective from the International Clearinghouse for Birth Defects Surveillance and Research. *American Journal of Public Health*.

[17] Monlleo IL, Mossey PA, Gil-da-Silva-Lopes VL (2009). Evaluation of craniofacial care outside the brazilian reference network for craniofacial treatment. *The Cleft Palate-Craniofacial Journal*.

[18] World Health Organisation (WHO) (2003). *Global Registry and Database on Craniofacial Anomalies*.

[19] World Health Organisation (WHO) (2006). *Addressing the Global Challenges of Craniofacial Anomalies*.

[20] Shaw W (2004). Global strategies to reduce the health-care burden of craniofacial anomalies: report of WHO meetings on International. Collaborative Research on Craniofacial Anomalies. *The Cleft Palate-Craniofacial Journal*.

[21] Monlleó IL, Gil-da-Silva-Lopes VL (2006). Anomalias craniofaciais: descricao e avaliacao das caracteristicas gerais da atencao no Sistema Unico de Saude. *Cad Saude Publica*.

[22] Monlleó IL, Gil-Da-Silva-Lopes VL (2006). Brazil’s Craniofacial project: genetic evaluation and counseling in the reference network for craniofacial treatment. *The Cleft Palate-Craniofacial Journal*.

[23] Righeto ALC, Huber J, Machado JC, Melo DG (2008). Anomalias congênitas: validade das informações das declarações de nascido vivo em uma maternidade de Ribeirão Preto, São Paulo. *Pediatria*.

[24] Geremias AL, Almeida MF, Flores MPO (2009). Avaliação das Declarações de Nascido Vivo como fonte de informações sobre defeitos congênitos. *Revista Brasileira de Epidemiologia*.

[25] Luquetti DV, Koifman RJ (2009). Quality of reporting on birth defects in birth certificates: case study from a Brazilian reference hospital. *Cadernos de Saude Publica*.

[26] World Health Organization (WHO) Programmes and projects. http://www.who.int/genomics/anomalies/americas_registry/en/index.html.

[27] Monlleó IL (2008). *Atenção a pessoas com anomalias craniofaciais no Brasil: avaliação e propostas para o Sistema Único de Saúde*.

[28] Monlleó IL, Mossey PA, Gil-da-Silva-Lopes VL The Brazilian database on orofacial clefts: preliminary validation.

[29] Instituto Brasileiro de Geografia e Estatistica (IBGE) http://www.censo2010.ibge.gov.br/sinopse/index.php?dados=4&uf=00.

[30] Horovitz DDG, Llerena JC, Mattos RA (2006). Atenção aos defeitos congênitos no Brasil: características do atendimento e propostas para formulação de políticas públicas em genética clínica. *Cadernos de Saúde Pública*.

[31] Fontes MIB, Almeida LN, Reis GO (2012). Local strategies to address health needs of individuals with orofacial clefts in Alagoas, Brazil. *The Cleft Palate-Craniofacial Journal*.

[32] FitzPatrick DR, Raine PAM, Boorman JG (1994). Facial clefts in the west of Scotland in the period 1980–1984: epidemiology and genetic diagnoses. *Journal of Medical Genetics*.

[33] Shaw GM, Carmichael SL, Yang W, Harris JA, Lammer EJ (2004). Congenital malformations in births with orofacial clefts among 3.6 million California births, 1983–1997. *American Journal of Medical Genetics*.

[34] Freire-Maia N (1957). Inbreeding in Brazil. *American Journal of Human Genetics*.

[35] Freire-Maia N (1990). Genetic effects in Brazilian populations due to consanguineous marriages. *American Journal of Medical Genetics*.

[36] Freire-Maia N (1990). Consanguinity marriages in Brazil. *Revista Brasileira de Biologia*.

[37] Harville EW, Wilcox AJ, Lie RT, Vindenes H, Åbyholm F (2005). Cleft lip and palate versus cleft lip only: are they distinct defects?. *American Journal of Epidemiology*.

[38] Grosen D, Chevrier C, Skytthe A (2010). A cohort study of recurrence patterns among more than 54000 relatives of oral cleft cases in Denmark: support for the multifactorial threshold model of inheritance. *Journal of Medical Genetics*.

[39] Rahimov F, Marazita ML, Visel A (2008). Disruption of an AP-2*α* binding site in an IRF6 enhancer is associated with cleft lip. *Nature Genetics*.

[40] Herkrath AP, Herkrath FJ, Rebelo MA, Vettore MV (2012). Parental age as a risk factor for non-syndromic oral clefts: a meta analysis. *Journal of Dentistry*.

[41] Strauss RP, Broder H (1993). Children with cleft lip/palate and mental retardation: a subpopulation of cleft-craniofacial team patients. *The Cleft Palate-Craniofacial Journal*.

[42] Online Mendilian Inheritance in Man (OMIM) Syndromes with cleft lip and or cleft palate. http://www.ncbi.nlm.nih.gov/omim.

[43] World Health Organization (WHO) (2006). *Medical Genetic Services in Developing Countries. the Ethical, Legal and Social Implications of Genetic Testing and Screening*.

[44] Daar AS, Berndtson K, Persad DL, Singer PA (2007). How can developing countries harness biotechnology to improve health?. *BMC Public Health*.

[45] Kohonen-Corish MR, Al-Aama JY, Auerbach AD (2010). Human Variome Project Meeting. How to catch all those mutations—the report of the third Human Variome Project Meeting, UNESCO Paris, May 2010. *Human Mutation*.

[46] Rasmussen SA, Olney RS, Holmes LB, Lin AE, Keppler-Noreuil KM, Moore CA (2003). Guidelines for case classification for the National Birth Defects Prevention Study. *Birth Defects Research A*.

